# P-202. Predominance of DENV-3 circulation in the main private medical center in an urban region of Miranda, Venezuela

**DOI:** 10.1093/ofid/ofaf695.424

**Published:** 2026-01-11

**Authors:** Natasha A Camejo-Avila, Zoila A Moros, Óscar Omaña-Ávila, Angel Navia-Padilla, Oriana F Balducci-Di Mattia, Martin Carballo, Celina Elster, David Forero-Peña, José A Zambrano

**Affiliations:** Universidad Central de Venezuela, Caracas, Miranda, Venezuela; Venezuelan Institute for Scientific Research, Caracas, Miranda, Venezuela; Universidad Central de Venezuela, Caracas, Miranda, Venezuela; Universidad Central de Venezuela, Caracas, Miranda, Venezuela; Universidad Central de Venezuela, Caracas, Miranda, Venezuela; Universidad Central de Venezuela, Caracas, Miranda, Venezuela; Laboratory of Microbiology, Centro médico Docente la trinidad, Miranda, Venezuela., Caracas, Distrito Federal, Venezuela; Biomedical Research and Therapeutic Vaccines Institute, Ciudad Bolívar, Venezuela, Caracas, Distrito Federal, Venezuela; Venezuelan Institute for Scientific Research, Caracas, Miranda, Venezuela

## Abstract

**Background:**

The global incidence of dengue virus (DENV) has increased dramatically over the past two decades, rising from 500,000 cases to 5.2 million cases between 2000 and 2019, and spreading across 129 countries. 2024 marked a turning point in the history of public health in the Americas, as the region faced an unprecedented increase in dengue cases, with 13,034,758 reported cases, 22,724 severe cases, and 8,244 deaths. In Venezuela, a more than 9-fold increase in cases has been reported in the last 25 years, however, the last report from the Ministry of Health was published in 2016. There are no recent studies that epidemiologically characterize the infection and show the circulating serotypes of the virus.

**Figure:**
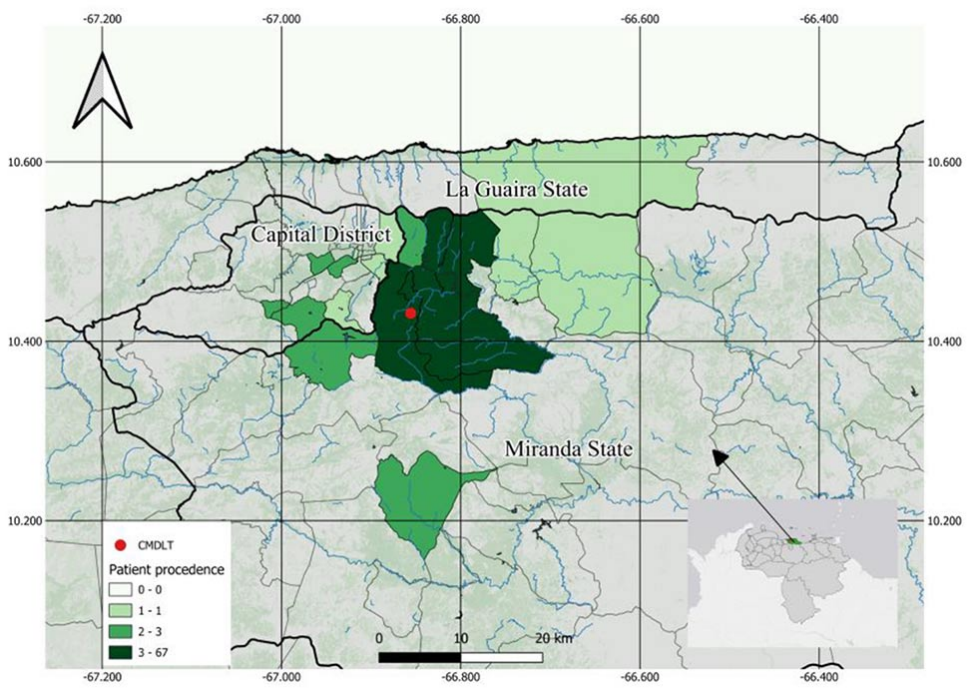
Map of distribution of cases by parish

Patient origin map with the location of the medical private center (Red).

**Methods:**

We conducted a cross-sectional study to identify the epidemiological, clinical, and molecular behavior of dengue in screened patients in a private health care center in Miranda, Venezuela. From March to November 2024, samples were collected from patients with febrile syndrome attended in one of the main clinics of the city, a total of 200 febrile patients were serologically confirmed for dengue infection by IgG/IgM and/or NS1 immunochromatographic rapid tests. Using real-time reverse transcriptase polymerase chain reaction, 160 patients were serotyped, the DENV envelope protein gene was sequenced, and the predominant genotype was determined by phylogenetic and phylogeographic analysis.

**Results:**

The median age was 37 years with an age range between 3 and 88 years, the majority were men (54.3%), and they were mainly from Miranda (88.1%). Most cases occurred in the age range of 18 to 40 years (25.6%), followed by those younger than 18 years (23.7%), in a smaller proportion older than 65 years (15.6%). Cocirculation of the four serotypes was observed. Serotype DENV-3 predominated in 153 of160 samples (95%), DENV-2 was identified in 4 samples, DENV-4 and DENV-1 in one sample each and co-infection of 2 serotypes was documented in one patient (DENV-3 and DENV-4). Serotypes did not correlate with disease severity.

**Conclusion:**

This study provides updated information on the clinical, virological, and demographic aspects of dengue disease in an urban region of Caracas, Venezuela.

**Disclosures:**

All Authors: No reported disclosures

